# Seizure- or Epilepsy-Related Emergency Department Visits Before and During the COVID-19 Pandemic — United States, 2019–2021

**DOI:** 10.15585/mmwr.mm7121a2

**Published:** 2022-05-27

**Authors:** Sanjeeb Sapkota, Elise Caruso, Rosemarie Kobau, Lakshmi Radhakrishnan, Barbara Jobst, Jourdan DeVies, Niu Tian, R. Edward Hogan, Matthew M. Zack, Daniel M. Pastula

**Affiliations:** ^1^Office of the Director, Center for Global Health, CDC; ^2^Division of Tuberculosis Elimination, National Center for HIV/AIDS, Viral Hepatitis, STD, and TB Prevention, CDC; ^3^Division of Population Health, National Center for Chronic Disease Prevention and Health Promotion, CDC; ^4^Division of Health Informatics and Surveillance, Center for Surveillance, Epidemiology, and Laboratory Services, CDC; ^5^Geisel School of Medicine, University of Dartmouth, Hanover, New Hampshire; ^6^ICF International, Fairfax, Virginia; ^7^Washington University, St. Louis, Missouri; ^8^University of Colorado School of Medicine and Colorado School of Public Health, Aurora, Colorado.

Seizures, transient signs or symptoms caused by abnormal surges of electrical activity in the brain, can result from epilepsy, a neurologic disorder characterized by abnormal electrical brain activity causing recurrent, unprovoked seizures, or from other inciting causes, such as high fever or substance abuse (*1*). Seizures generally account for approximately 1% of all emergency department (ED) visits (*2**,**3*). Persons of any age can experience seizures, and outcomes might range from no complications for those with a single seizure to increased risk for injury, comorbidity, impaired quality of life, and early mortality for those with epilepsy (*4*). To examine trends in weekly seizure- or epilepsy-related (seizure-related) ED visits^†^ in the United States before and during the COVID-19 pandemic, CDC analyzed data from the National Syndromic Surveillance Program (NSSP).^§^ Seizure-related ED visits decreased abruptly during the early pandemic period. By the end of 2020, seizure-related ED visits returned almost to prepandemic levels for persons of all ages, except children aged 0–9 years. By mid-2021, however, this age group gradually returned to baseline as well. Reasons for the decrease in seizure-related ED visits in 2020 among all age groups and the slow return to baseline among children aged 0–9 years compared with other age groups are unclear. The decrease might have been associated with fear of exposure to COVID-19 infection in EDs deterring parents or guardians of children from seeking care, adherence to mitigation measures including avoiding public settings such as EDs, or increased access to telehealth services decreasing the need for ED visits (*5*). These findings reinforce the importance of understanding factors associated with ED avoidance among persons with epilepsy or seizure, the importance that all eligible persons be up to date^¶^ with COVID-19 vaccination, and the need to encourage persons to seek appropriate care for seizure-related emergencies** to prevent adverse outcomes.

NSSP collects deidentified electronic health record data from EDs and other health care settings. ED visit data are derived from a subset of approximately 71% of the nation’s nonfederal EDs (i.e., EDs not supported by the Veterans Health Administration or U.S. Department of Defense). Diagnosis codes from the *International Classification of Diseases, Ninth Revision*, *Clinical Modification* (ICD-9-CM) and *International Classification of Diseases, Tenth Revision*, *Clinical Modification* (ICD-10-CM), Systematized Nomenclature of Medicine, and relevant free-text reason for visit (chief complaint) terms were used to identify seizure-related ED visits (Supplementary Table, https://stacks.cdc.gov/view/cdc/117412) (Supplementary Box, https://stacks.cdc.gov/view/cdc/117573). All analyses were restricted to EDs that reported consistently more complete data throughout the study period (January 1, 2019–December 31, 2021); 56% of EDs sharing data with NSSP met these criteria.^††^ CDC assessed trends by six age groups (0–9, 10–19, 20–39, 40–59, 60–69, and ≥70 years) and visualized age-specific trends of weekly seizure-related ED visits during 2019–2021. Using R (version 4.1.2; The R Foundation), CDC quantified change in mean weekly seizure-related ED visits during April 1–December 29 across 3 years: 2019, 2020, and 2021; results were stratified by age group and sex. Percentage change in mean weekly seizure-related ED visits was assessed by comparing 2020 data with corresponding data from 2019 and 2021. This activity was reviewed by CDC and was conducted consistent with applicable federal law and CDC policy.^§§^

All ED visits, including seizure-related ED visits, decreased among all age groups and among both males and females during the pandemic period April 1–December 29, 2020, compared with the corresponding period in 2019 (Table). The largest decline in seizure-related ED visits, noted as early as February 2020, was observed among children aged 0–9 years (Figure 1) (Figure 2). During April 1–December 29, 2020, the number of weekly seizure-related ED visits declined by 16% overall to 19,824, from 23,588 during the same period^¶¶^ in 2019 (Table). Among children aged 0–9 years, the number of seizure-related weekly ED visits declined by 44% to 1,553, compared with 2,759 visits during the same period in 2019; overall ED visits among children aged 0–9 years declined by 56%, from 162,711 visits in 2019 to 71,131 in 2020. By the first week of 2021, the number of seizure-related ED visits among all age groups was close to respective prepandemic levels in 2019, with the exception of children aged 0–9 years, among whom the rebound to prepandemic levels was delayed until approximately week 25 of 2021 (Figure 1). To examine whether the decrease among children aged 0–9 years was associated with pediatric febrile seizure burden, a posthoc analysis was conducted. In children aged 0–9 years, febrile seizures accounted for approximately one third of all seizure-related ED visits in all 3 years (approximately 35%, 31%, and 33% in 2019, 2020, and 2021, respectively).

**TABLE:** Mean weekly seizure- or epilepsy-related emergency department visits and overall emergency department visits, by age and sex, and percentage change* — National Syndromic Surveillance Program,^†^ United States, April 1–December 29, 2019–2021

Characteristic	Mean weekly visits, no. (95% CI)^§^			% Change	
	2019	2020	2021	2019–2020	2020–2021
**Seizure or epilepsy ED visits**					
**Age group, yrs**					
0–9	2,759 (2,660–2,864)	1,553 (1,504–1,593)	2,528 (2,462–2,593)	−44	63
10–19	1,893 (1,846–1,940)	1,413 (1,356–1,469)	1,749 (1,710–1,786)	−25	24
20–39	7,102 (7,037–7,165)	6,143 (5,957–6,316)	6,579 (6,478–6,680)	−13	7
40–59	6,476 (6,412–6,539)	5,701 (5,548–5,838)	5,769 (5,678–5,860)	−12	1
60–69	2,588 (2,561–2,617)	2,423 (2,373–2,467)	2,495 (2,468–2,524)	−6	3
≥70	2,641 (2,604–2,679)	2,504(2,441–2,561)	2,583 (2,557–2,613)	−5	3
**Sex**					
Female	11,422 (11,344–11,501)	9,327 (9,044–9,579)	10,373 (10,280–10,470)	−18	11
Male	12,128 (12,039–12,236)	10,462 (10,214–10,694)	11,387 (11,296–11,470)	−14	9
**Total**	**23,588 (23,429–23,739)**	**19,824 (19,295–20,311)**	**21,800 (21,614–21,969)**	**−16**	**10**
**All-cause ED visits**					
**Age group, yrs**					
0–9	162,711 (154,767–171,195)	71,131 (67,015–74,824)	142,868 (137,805–147,822)	−56	101
10–19	127,264 (123,781–130,677)	79,594 (74,870–84,171)	114,353 (111,036–117,884)	−37	44
20–39	416,652 (413,210–420,159)	336,598 (322,674–348,693)	401,671 (394,081–409,796)	−19	19
40–59	347,606 (344,299–350,816)	288,453 (278,532–297,426)	337,317 (331,750–342,781)	−17	17
60–69	157,694 (156,596–158,946)	135,574 (130,804–139,547)	161,899 (160,116–163,865)	−14	19
≥70	231,619 (230,000–233,699)	193,202 (185,523–199,808)	231,799 (229,713–233,852)	−17	20
**Sex**					
Female	797,473 (791,101–804,433)	593,418 (568,244–615,384)	755,769 (745,392–766,769)	−26	27
Male	651,555 (646,948–656,594)	513,365 (494,989–530,303)	636,576 (627,504–646,651)	−21	24
**Total**	**1,451,717 (1,441,285–1,463,581)**	**1,109,069 (1,067,564–1,148,844)**	**1,395,349 (1,374,389–1,415,093)**	**−24**	**26**

**Abbreviation:** ED = emergency department.

* The percentage change in visits between the surveillance and reference periods (2019 [reference] versus 2020 [surveillance] and 2020 [reference] versus 2021 [surveillance]) was calculated as (ED visits during surveillance period – ED visits during reference period)/ED visits during reference period x 100%.

^†^ The National Syndromic Surveillance Program receives anonymized medical record information from approximately 71% of nonfederal EDs nationwide. To reduce artifactual impact from changes in reporting patterns, analyses were restricted to facilities with more consistent reporting of more complete data (coefficient of variation ≤40 and average weekly informative discharge diagnosis ≥75% complete during 2019–2021).

^§^ CIs were constructed using the percentile bootstrap method using 1,000 replicate samples of the weekly counts. CIs were formed using the 2.5th and 97.5th percentiles of the bootstrap distributions.

**FIGURE 1:**
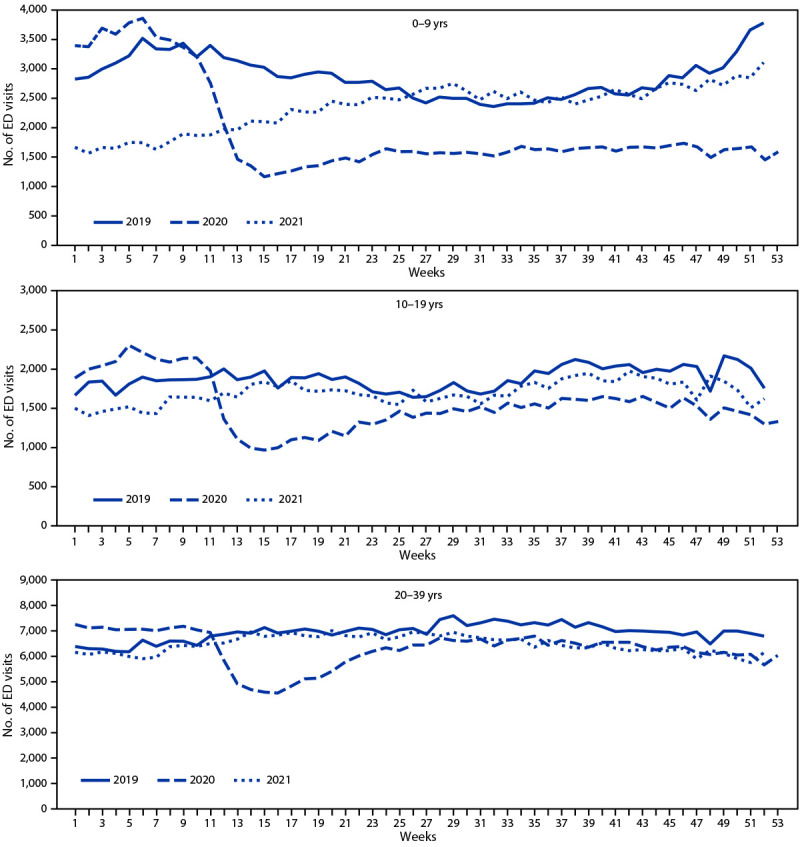
Weekly seizure- or epilepsy-related emergency department visits among persons aged <40 years, by age group* — National Syndromic Surveillance Program,^†^ United States, 2019–2021 **Abbreviation:** ED = emergency department. * The y-axis range differs for different age groups to account for different numbers of ED visits by these groups and to facilitate visualization of changes over time. ^†^ The National Syndromic Surveillance Program receives deidentified medical record information from approximately 71% of nonfederal EDs nationwide. To reduce artifactual impact from changes in reporting patterns, analyses were restricted to facilities with more consistent reporting of more complete data (coefficient of variation ≤40 and average weekly informative discharge diagnosis ≥75% complete during 2019–2021).

**FIGURE 2:**
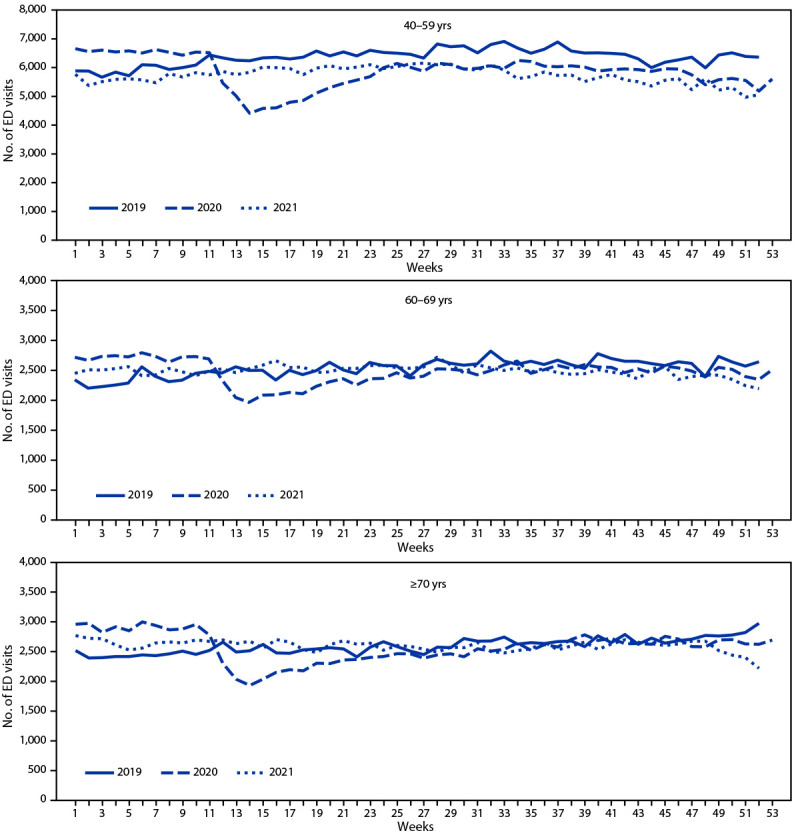
Weekly seizure- or epilepsy-related emergency department visits among persons aged ≥40 years, by age group* — National Syndromic Surveillance Program,^†^ United States, 2019–2021 **Abbreviation:** ED = emergency department. * The y-axis range differs for different age groups to account for different numbers of ED visits by these groups and to facilitate visualization of changes over time. ^†^ The National Syndromic Surveillance Program receives deidentified medical record information from approximately 71% of nonfederal EDs nationwide. To reduce artifactual impact from changes in reporting patterns, analyses were restricted to facilities with more consistent reporting of more complete data (coefficient of variation ≤40 and average weekly informative discharge diagnosis ≥75% complete during 2019–2021).

## Discussion

In this study of trends in seizure-related ED visits during the COVID-19 pandemic, seizure-related ED visits during the initial COVID-19 waves declined among all age groups, especially among children aged 0–9 years. These findings are consistent with several other studies (*6**–**8*). In one analysis of U.S. ED visits during January 2019–May 2020, the number of weekly all-cause ED visits declined abruptly during March 29–April 25, 2020, along with a decline in ED visits among children aged 0–9 years attributable to common conditions, including influenza, otitis media, upper respiratory conditions, asthma, viral infection, respiratory symptoms, and fever (*6*). International studies have described a reduction in seizure-related ED visits among children during the COVID-19 pandemic, with one study reporting a notable decline in febrile seizure–related ED visits among children aged 0–6 years (*7*,*8*).

The percentages of ED visits attributable to febrile seizures among children aged 0–9 years in this study were relatively stable, therefore any changes in ED visits for febrile seizures during the study period were unlikely to explain the overall change of trend in seizure-related ED visits in this age group. Researchers in Italy examined selected causes for seizure-related ED visits during February 23–April 21, 2020 (e.g., first episode or breakthrough seizure), but could not attribute the observed decrease in seizure-related ED visits to seizure type (e.g., febrile versus first episode seizures) (*7*). However, a limitation of the Italian study was small sample size; thus, the findings warrant additional study. The findings related to febrile seizure–attributable ED use in the current report differ from, but supplement growing research in this area (*8*).

In the present study, school closures and the need to shelter at home could have facilitated heightened supervision of children while at home, including increased monitoring and promotion of healthful behaviors reducing seizure risk (e.g., medication adherence and regular sleep) or seizure sequelae (e.g., injury), thereby reducing the need for ED care (*7*,*9*). The decrease in weekly seizure-related ED visits among children aged 0–9 years might also have been associated with concern about risk for COVID-19 in EDs, deterring parents or guardians from seeking care for their children. It is also possible that expanded access and increased use of telehealth facilitated triaged telephone support or virtual health care encounters, especially for children with epilepsy and high-risk comorbidities, otherwise obtained in EDs (*5**,**10*). Additional studies are warranted to determine whether decreased in-person ED care for children with seizures or epilepsy during the initial COVID-19 pandemic was associated with any differences in risk for infection, injury, or delayed care, seizure type, or other factors and any associations between these factors and adverse outcomes.

The findings in this report are subject to at least four limitations. First, because NSSP coverage varies both within and across states, NSSP data are not nationally representative. In some states nearly all hospitals report, while in others only those in certain counties or health care systems report. Thus, these findings might not be generalizable. Second, differences in availability, coding practices, and reporting of chief complaints and discharge diagnoses from facilities might influence trends. To limit the impact of changing data volume and underlying data quality on results, only data from hospitals with consistent reporting and more complete data were included in this analysis. Third, trends displayed are restricted to ED visits only, and do not capture treatment sought for seizures in other settings. Finally, distinguishing initial seizure-related visits from subsequent visits was not possible, therefore the numbers of ED visits reported might represent multiple visits by one person.

These findings reinforce the importance of understanding factors associated with ED avoidance among persons with epilepsy or seizures, and any alternative care approaches among persons with epilepsy or seizures and the need to encourage persons to seek appropriate care for seizure-related emergencies. Vaccination against SARS-CoV-2, the virus that causes COVID-19, of all age-eligible persons, including those with epilepsy, is recommended to protect against the adverse effects of COVID-19 (*9*).

**Summary**

**What is already known about this topic?**

Seizures or epilepsy account for 1% of annual emergency department (ED) visits. Data on seizure- or epilepsy-related ED visits during the COVID-19 pandemic are limited.

**What is added by this report?**

Weekly seizure- or epilepsy-related ED visits decreased sharply during the early pandemic period among all age groups, especially children aged 0–9 years. The return to prepandemic baseline in this group was delayed until mid-2021, longer than other age groups.

**What are the implications for public health practice?**

These findings reinforce the importance of understanding factors associated with ED avoidance among persons with epilepsy or seizure, the importance that all eligible persons be up to date with COVID-19 vaccination, and the need to encourage persons to seek appropriate care for seizure-related emergencies.

## Footnotes

* Deceased.

† Analysis was limited to ED encounters. As of December 31, 2021, the median number of facilities included in the analysis was 2,031 (range = 1,986–2,038), including data from 56% of all nonfederal EDs sharing data with NSSP.

§ NSSP is a collaboration among CDC, federal partners, local and state health departments, and academic and private sector partners. NSSP receives deidentified electronic health data from 50 states representing approximately 71% of nonfederal EDs nationwide, although <50% of ED facilities from California, Hawaii, Iowa, Minnesota, Ohio, and Oklahoma currently participate in NSSP at the time of this analysis.

¶
https://www.cdc.gov/coronavirus/2019-ncov/vaccines/stay-up-to-date.html

** Includes a first-time seizure and status epilepticus, which is defined as a continuous seizure lasting >5 minutes or recurrent seizures without regaining consciousness between seizures.

†† To limit the impact of data quality on trends, all analyses were restricted to facilities with a coefficient of variation ≤40 and percentage of weekly average informative discharge diagnosis ≥75 throughout the analysis period (January 2019–December 2021) so that only consistently reporting facilities with more complete data were included. EDs that met these data quality control criteria were included in the analysis.

§§ 45 C.F.R. part 46.102(l)(2), 21 C.F.R. part 56; 42 U.S.C. Sect. 241(d); 5 U.S.C. Sect. 552a; 44 U.S.C. Sect. 3501 et seq.

¶¶ Percentage change in visits during surveillance periods compared with reference periods (surveillance period April 1–December 29, 2020, compared with reference period April 1–December 29, 2019, and surveillance period April 1–December 29, 2021, compared with reference period April 1–December 29, 2020) was calculated as (ED visits for seizures or epilepsy during surveillance period – ED visits for seizures or epilepsy during reference period)/ED visits for seizures or epilepsy during reference period x 100%.
